# Evaluations of biomarkers associated with 5-FU sensitivity for non-small-cell lung cancer patients postoperatively treated with UFT

**DOI:** 10.1038/sj.bjc.6603297

**Published:** 2006-08-01

**Authors:** J Nakano, C Huang, D Liu, D Masuya, T Nakashima, H Yokomise, M Ueno, H Wada, M Fukushima

**Affiliations:** 1 Faculty of Medicine, Second Department of Surgery, Kagawa University, 1750-1, Miki-cho, Kita-gun, Kagawa 761-0793, Japan; 2 Faculty of Medicine, Department of Pathology and Host Defense, Kagawa University, Kagawa, Japan; 3 Faculty of Medicine, Department of Thoracic Surgery, Kyoto University, 54, Shogoin-kawahara-cho, Sakyo-ku, Kyoto, 606-8397, Japan; 4 Pathology Optimal Medication Research Laboratory, Taiho Pharmaceutical Co. Ltd, 224-2, Hiraishi-Ebisuno, Kawauchi-cho, Tokushima-shi, Tokushima 771-0132, Japan

**Keywords:** lung cancer, 5-FU, thymidylate synthase, orotate phosphoribosyltransferase, dihydropyrimidine dehydrogenase

## Abstract

The sensitivity to 5-fluorouracil (5-FU) has been reported to be associated with target molecule thymidylate synthase (TS), fluoropyrimidine-metabolising enzymes such as orotate phosphoribosyltransferase (OPRT), and dihydropyrimidine dehydrogenase (DPD). We performed an immunohistochemical study on the clinical significance of TS, OPRT, and DPD expression using 151 resected non-small-cell lung cancer (NSCLC) patients postoperatively treated with a combination of tegafur and uracil (UFT). Eighty-two carcinomas were TS-positive, 105 carcinomas were OPRT-positive, 68 carcinomas were DPD-positive. No correlation was observed in the HSCORE between the TS and OPRT expression (*r*=0.203), between the TS and DPD expression (*r*=0.098), or between the OPRT and DPD expression (*r*=0.074). Regarding the survival of NSCLC patients treated with UFT, the 5-year survival rate of patients with TS-negative tumours was significantly higher than that with TS-positive tumours (*P*=0.0133). The 5-year survival rate of patients with OPRT-positive stage II to III tumours was significantly higher than that with OPRT-negative stage II to III tumours (*P*=0.0145). In addition, the 5-year survival rate of patients with DPD-negative tumours was also significantly higher than that with DPD-positive tumours (*P*=0.0004). A Cox multivariate regression analysis revealed the TS status (hazard ratio 2.663; *P*=0.0003), OPRT status (hazard ratio 2.543; *P*=0.0005), and DPD status (hazard ratio 2.840; *P*<0.0001) to all be significant prognostic factors for the survival of resected NSCLC patients postoperatively treated with UFT.

Lung cancer is one of the most common cancers and the major cause of cancer-related death in North America, Europe, and Japan (Cersosimo, 2002). Non-small-cell lung cancer (NSCLC) comprises about 75% of all lung cancers. The majority of NSCLC patients have locally advanced stage III or metastatic stage IV disease at diagnosis. In addition, even in the early stage, some NSCLC patients may experience recurrence after surgery. Therefore, new therapeutic strategies are required to improve the clinical outcome of NSCLC patients.

Both experimental and clinical studies based on the molecular biology have revealed many molecules to affect various biological behaviors of malignant tumours including NSCLCs. To improve the treatment of NSCLC patients, it is important to design an optimal therapeutic strategy according to tumour biology (Huang *et al*, 2005). Therefore, the selection of effective adjuvant therapies based on biomarkers associated with tumour biology, such as made-to-order chemotherapy, is considered to be an effective strategy for NSCLC patients (Rosell *et al*, 2003; Vokes and Choy, 2003). Thus, the development of highly specific and responsive sensitivity tests for anticancer agents is important to be able to predict the response to an anticancer agent before using it for treatment. Recent experimental and clinical studies have reported several molecular targeted therapies, such as 5-fluorouracil (5-FU)-derived agents against thymidylate synthase (TS) (Rustum *et al*, 1997), Gefitinib or Erlotinib against epidermal growth factor receptor (Mitsudomi *et al*, 2005), and Bevacizumab against vascular endothelial growth factor-A (Johnson *et al*, 2004).

Among them, 5-FU-derived agents have been widely used in the treatment of various human cancers, including NSCLCs (Wada *et al*, 1996; Kato *et al*, 2004), colorectal cancers (Macdonald and Astrow, 2001), and gastric cancers (Takiuchi and Ajani, 1998), since its introduction into clinical practice more than 40 years ago. Clinical studies have reported their sensitivity to be associated with the target molecule TS (Rustum *et al*, 1997), fluoropyrimidine-metabolising enzymes such as orotate phosphoribosyltransferase (OPRT) (Peters *et al*, 1986), and the 5-FU-catabolitic enzyme such as dihydropyrimidine dehydrogenase (DPD) (Beck *et al*, 1994). However, there were only a few clinical studies on the simultaneous evaluations of these biomarkers to evaluate 5-FU sensitivity (Isshi *et al*, 2002; Fujii *et al*, 2003; Ichikawa *et al*, 2003a).

Therefore, we performed a clinical study on the clinical significance of TS, OPRT, and DPD expression for the treatment of resected NSCLC patients who were postoperatively treated with oral 5-FU derivatives, UFT (a combination of tegafur and uracil, Taiho Pharmaceutical Co., Tokyo, Japan) (Wada *et al*, 1996; Kato *et al*, 2004).

## MATERIALS AND METHODS

### Clinical characteristics of patients

From January 1995 to December 2000, consecutive NSCLC patients who underwent surgery and were postoperatively treated with UFT, at the Second Department of Surgery, Faculty of Medicine, Kagawa University, were studied. This study was approved by the institutional review board of Kagawa University (14-7, a clinical study of biological markers in NSCLCs) and signed informed consent was obtained from each patient. Tumour node metastasis staging designations were made according to the postsurgical pathological international staging system (Mountain, 1997). In total, 151 patients with lung cancer up to stage III were investigated (Table 1). They included 62 patients with stage I NSCLCs, 28 patients with stage II NSCLCs, and 61 patients with stage III NSCLCs. The patients' clinical records and histopathological diagnoses were fully documented. This report includes follow-up data as of 31 October 2005. The median follow-up period for all patients was 65.7 months.

Regarding the methods for a surgical resection, a pneumonectomy was performed in 14 patients with stage II to III NSCLCs. A lobectomy was performed in 123 patients; in 48 patients with stage I NSCLCs, in 26 patients with stage II NSCLCs, and in 49 patients with stage III NSCLCs. A segmentectomy was performed in four patients with stage I NSCLCs, and a wedge resection was performed in 10 patients with stage I NSCLCs. Platinum-based chemotherapy using mitomycin/vinblastin/cisplatin or carboplatin/paclitaxel was performed in 79 patients with stage II to III NSCLCs; neoadjuvant chemotherapy in 39 patients, and postoperative adjuvant chemotherapy in 40 patients with nodal metastases. Radiation therapy was performed in 24 patients; in 17 patients with T3 or T4 status and in seven patients with mediastinal lymph node metastases. The oral administration of UFT (300–400 mg day^−1^ body^−1^) was started within 1 month after surgery. UFT was administered for 2 years if there was no recurrence of carcinomas. In patients with recurrences, UFT was given until its oral administration became impossible.

### Immunohistochemistry

Formalin-fixed paraffin-embedded tissue was cut into 4-*μ*m sections and mounted on poly-L-lysine-coated slides. The sections were deparaffinised and rehydrated. The slides were then heated in a microwave for 10 min in a 10-*μ*mol l^−1^ citrate buffer solution at pH 6.0, and cooled to room temperature for 20 min. After quenching the endogenous peroxidase activity with 0.3% H2O2 (in absolute methanol) for 30 min, the sections were treated for 2 h at room temperature with 5% bovine serum albumin to block nonspecific staining. Duplicate sections were incubated overnight with the primary specific antibodies detecting TS (rabbit IgG, diluted at 1 : 500), OPRT (rabbit IgG, diluted at 1 : 1000), and DPD (rabbit IgG, diluted at 1 : 500) (Huang *et al*, 2000). The slides were then incubated for 1 h with biotinylated anti-rabbit IgG (Vector Laboratories Inc., Burlingame, CA, USA). The sections were incubated with the avidin–biotin–peroxidase complex (Vector Laboratories Inc.) for 1 h, and antibody binding was visualised with 3,3′-diaminobenzidine tetrahydrochloride. Finally, the sections were lightly counterstained with Mayer's hematoxylin. The human colon cancer cell line DLD-1/FrUrd was used as a positive control for the staining of TS. Sections of resected lung tumours to express OPRT were used as positive controls for the staining of OPRT. The human pancreatic cancer cell line MIAPaCa-2 was used as a positive control for the staining of DPD. A rabbit IgG was used as a negative control during each run of immunohistochemical staining.

All of the immunostained sections were reviewed by two authors (J Nakano and M Ueno) who had no knowledge of the patients' clinical status. In cases of multiple areas of low intensity, five areas selected at random were scored. In sections where all of the staining appeared to be intense, one random field was selected. At least 200 tumour cells were scored per × 40 field. All sections were scored in a semiquantitative manner according to a previously described method, which reflects both the intensity and percentage of cells staining at each intensity (McCarty *et al*, 1986). Intensity was classified as 0 (no staining), +1 (weak staining), +2 (distinct staining), or +3 (very strong staining). A value designated as the ‘HSCORE’ was obtained for each slide by using the following algorithm: HSCORE=∑(*I* × PC), where *I* and PC represent the staining intensity and the percentage of cells that stain at each intensity, respectively. And the corresponding HSCOREs were calculated separately. Concerning TS expression, when the HSCORE of TS in a given specimen was ⩾30, the sample was classified as TS-positive, the same as reported previously (Huang *et al*, 2000). Concerning OPRT expression, the sample was classified as OPRT-positive when the HSCORE of OPRT in a given specimen was ⩾30, because the cutoff line of 30 showed the most significant association with a survival difference. Concerning DPD expression, when the HSCORE of DPD in a given specimen was ⩾50, the sample was classified as DPD-positive, the same as reported previously (Huang *et al*, 2000).

### Statistical analysis

The statistical differences in the expression of each biological marker in relation to various clinical and pathological parameters were assessed by the *χ*^2^-test. Overall survival was defined as the time from treatment initiation (surgical resection, chemotherapy, or radiation) to the date of death from any cause. The Kaplan–Meier method was used to estimate the probability of overall survival as a function of time, and differences in the survival of subgroups of patients were compared by using Mantel's log-rank test. A multivariate analysis was performed using the Cox regression model to study the effects on survival (Cox, 1972). All *P*-values were based on two-tailed statistical analysis, and a *P*-value <0.05 was considered to indicate statistical significance.

## RESULTS

### Clinical significance of intratumoural TS expression in NSCLCs treated with UFT

**Figure 1:**
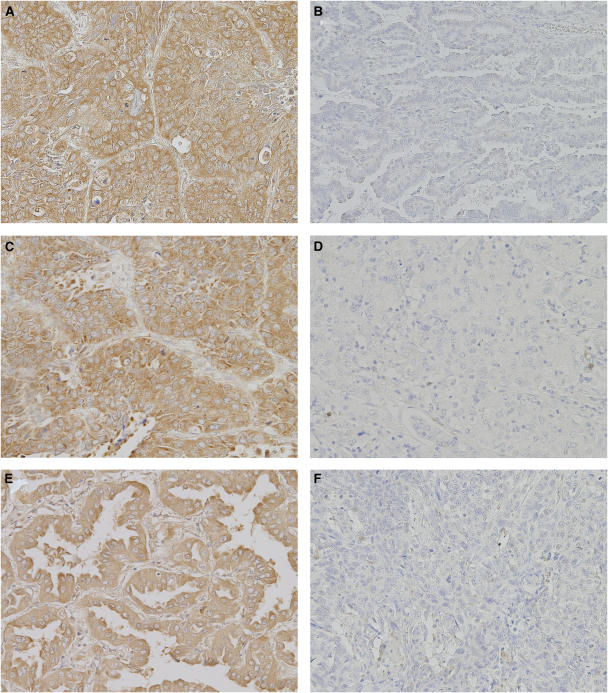
Immunohistochemical staining of human NSCLC tissues (original magnification, × 100). (**A**, **B**) Carcinoma with positive and negative TS expression, (**C**, **D**) carcinoma with positive and negative OPRT expression, (**E**, **F**) carcinoma with positive and negative DPD expression.

The intratumoural TS expression appeared in a cytoplasmic staining pattern (Figure 1A and 1B). Of the 151 NSCLCs we studied, 82 carcinomas (54.3%) were TS-positive, and 69 carcinomas (45.7%) were TS-negative (Table 2). The frequency of TS-positive tumours was significantly higher in squamous cell carcinomas than in adenocarcinomas (69.6 *vs* 42.9%, *P*=0.0063). However, there was no difference in the TS status according to tumour status, nodal status, pathological stage, tumour differentiation, or neoadjuvant chemotherapy.

**Figure 2:**
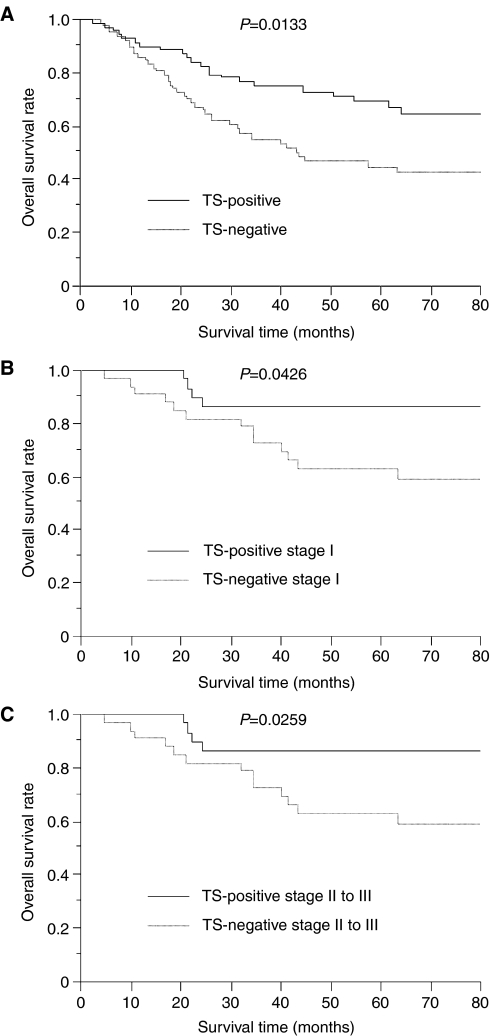
Overall survival of NSCLC patients treated UFT in relation to TS status. (**A**) Total NSCLCs. (**B**) Stage I NSCLCs. (**C**) Stage II to III NSCLC.

Regarding the survival of NSCLC patients treated with UFT, the 5-year survival rate of patients with TS-negative tumours was significantly higher than that with TS-positive tumours (69.1 *vs* 44.7%, *P*=0.0133, Figure 2A). Regarding the pathological stage, the 5-year survival rate of patients with TS-negative stage I tumours was significantly higher than that with TS-positive stage I tumours (86.2 *vs* 63.2%, *P*=0.0426, Figure 2B). In addition, the 5-year survival rate of patients with TS-negative stage II to III tumours was also significantly higher than that with TS-positive stage II to III tumours (55.6 *vs* 27.9%, *P*=0.0259, Figure 2C).

### Clinical significance of intratumoural OPRT expression in NSCLCs treated with UFT

The intratumoural OPRT expression appeared in a cytoplasmic staining pattern (Figure 1C and D). Of the 151 NSCLCs, 105 carcinomas (69.5%) were OPRT-positive, and 46 carcinomas (30.5%) were OPRT-negative (Table 2). There was no difference in the OPRT status according to tumour histology, tumour status, nodal status, pathological stage, tumour differentiation, or neoadjuvant chemotherapy.

**Figure 3:**
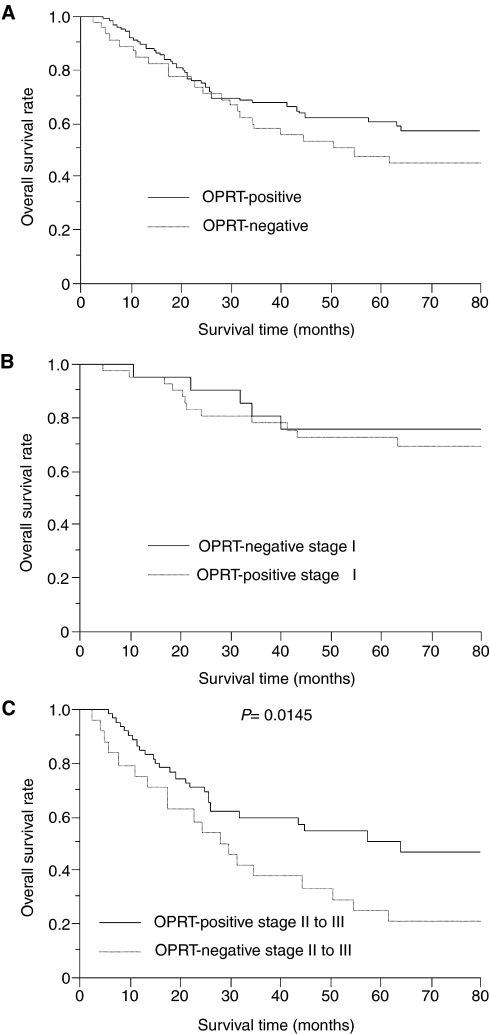
Overall survival of NSCLC patients treated UFT in relation to OPRT status. (**A**) Total NSCLCs. (**B**) Stage I NSCLCs. (**C**) Stage II to III NSCLC.

Regarding the survival of NSCLC patients treated with UFT, there was no significant difference in the 5-year survival rates between patients with OPRT-positive tumours and patients with OPRT-negative tumours (60.6 *vs* 48.0%, Figure 3A). Regarding the pathological stage, there was also no significant difference in the 5-year survival rates of patients with stage I NSCLCs, according to the OPRT status (Figure 3B). However, among locally advanced stage II to III, the 5-year survival rate of patients with OPRT-positive tumours was significantly higher than that with OPRT-negative tumours (50.8 *vs* 25.0%, *P*=0.0145, Figure 3C).

### Clinical significance of intratumoural DPD expression in NSCLCs treated with UFT

The intratumoural DPD expression appeared in a cytoplasmic staining pattern (Figure 1E and 1F). Of the 151 NSCLCs, 68 carcinomas (45.0%) were DPD-positive, and 83 carcinomas (55.0%) were DPD-negative (Table 2). There was no difference in the DPD status according to tumour histology, tumour status, nodal status, pathological stage, tumour differentiation, or neoadjuvant chemotherapy.

**Figure 4:**
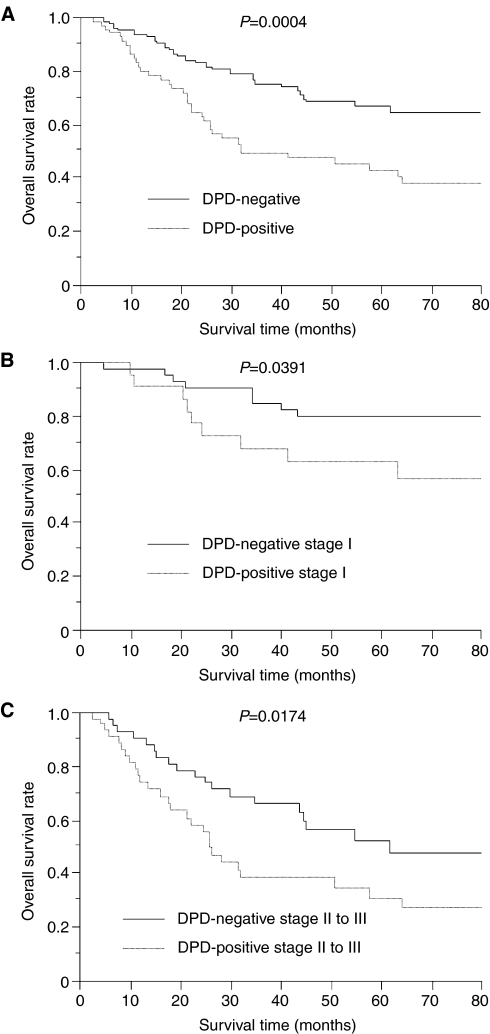
Overall survival of NSCLC patients treated UFT in relation to DPD status. (**A**) Total NSCLCs. (**B**) Stage I NSCLCs. (**C**) Stage II to III NSCLC.

Regarding the survival of NSCLC patients treated with UFT, the 5-year survival rate of patients with DPD-negative tumours was significantly higher than that with DPD-positive tumours (66.6 *vs* 42.6%, *P*=0.0004, Figure 4A). Regarding the pathological stage, the 5-year survival rate of patients with DPD-negative stage I tumours was significantly higher than that with DPD-positive stage I tumours (79.8 *vs* 63.0%, *P*=0.0391, Figure 4B). In addition, the 5-year survival rate of patients with DPD-negative stage II to III tumours was also significantly higher than that with DPD-positive stage II to III tumours (52.4 *vs* 31.0%, *P*=0.0174, Figure 4C).

### Prognostic factors of resected NSCLC patients treated with UFT

No correlation was observed in the HSCORE between the TS expression and OPRT expression (*r*=0.203), between the TS expression and DPD expression (*r*=0.098), or between the OPRT expression and DPD expression (*r*=0.074). A Cox multivariate regression analysis of the prognostic variables for resected NSCLC patients treated with UFT is shown in Table 3. Five variables, including the TS status (hazard ratio 2.663; *P*=0.0003), OPRT status (hazard ratio 2.534; *P*=0.0005), DPD status (hazard ratio 2.840; *P*<0.0001), pathological stage (hazard ratio 1.908; *P*<0.0001), and the methods of surgical resection (a pneumonectomy *vs* other methods)(hazard ratio 2.555; *P*=0.0242), were demonstrated to be significant prognostic factors for survival of resected NSCLC patients postoperatively treated with UFT.

## DISCUSSION

5-Fluorouracil is one of the most widely used antitumour chemothrapeutic agents for the treatment of a variety of human malignancies, especially in terms of the patient quality of life and economy (Wada *et al*, 1996). In fact, prospective randomised clinical studies have found the administration of UFT to be an effective adjuvant chemotherapy for NSCLC patients (Wada *et al*, 1996; Kato *et al*, 2004). On the other hand, many experimental and clinical studies have also revealed the 5-FU sensitivity to be affected by several biomarkers, such as its target molecule TS (Rustum *et al*, 1997), several fluoropyrimidine metabolising enzymes (Peters *et al*, 1986; Peters *et al*, 1991), and DPD, an enzyme of 5-FU catabolism (Beck *et al*, 1994).

First of all, regarding TS, its activity is necessary for cell proliferation because it catalyses the conversion of deoxyuridine-5′-monophosphate to deoxythymidine-5′-monophosphate, an essential step in DNA synthesis. After 5-FU is converted into 5-fluoro-2′-deoxyuridine 5′-monophosphate (FdUMP), this activated molecule forms a tight-binding complex with TS and 5,10-methylene-tetrahydrofolate, thus resulting in the inhibition of the TS activity (Rustum *et al*, 1997). Thymidylate synthase is therefore a target molecule of 5-FU. In addition, previous studies have reported that the regulation of the TS expression was affected by several mechanisms, including the polymorphism of the tandem repeated sequences in the promoter enhancer region of its gene and the genetic amplification of 18p (Horie *et al*, 1995; Hidaka *et al*, 2003).

In fact, many previous studies have reported the intratumoural TS expression to be associated with tumour proliferation (Navalgund *et al*, 1980; Nakagawa *et al*, 2004), the responsiveness to 5-FU (Johnston *et al*, 1995; Yeh *et al*, 1998). In addition, the overexpression of TS is reported to be associated with a poor prognosis of many cancer patients treated with 5-FU, including NSCLCs (Huang *et al*, 2000) and gastrointestinal cancers (Lenz *et al*, 1996). The present study in a relatively large number of patients treated with UFT also revealed that the survival of patients with TS-negative tumours was significantly better than that with TS-positive tumours. Furthermore, the significant differences in the survival of patients were observed in stage I cases as well as in locally advanced stage II to III cases. These results might be due to not only the responsiveness to 5-FU but also tumour proliferation (Nakagawa *et al*, 2004; Huang *et al*, 2005). In fact, recent studies have found that TS exhibits an oncogene-like activity (Rahman *et al*, 2004). In addition, the overexpression of TS itself is an independent factor for a poor prognosis in NSCLC patients (Huang *et al*, 2005).

Secondly, because 5-FU itself is a prodrug which requires its intracellular conversion, interindividual variation in the activity of fluoropyrimidine-metabolising enzymes can affect the activation of 5-FU. In general, phosphorylation is necessary to activate 5-FU by one or more of three pathways (Peters *et al*, 1986; Peters *et al*, 1991). Among them, OPRT is considered to be a major enzyme in the 5-FU conversion, directly to 5-fluorouridine 5′-monophosphate (FUMP) leading to the formation of FdUMP in the presence of 5-phosphoribosyl-1-pyrophosphate as a cofactor (Shirasaka *et al*, 1993). Experimental studies using cell lines have revealed a decreased expression and activity of OPRT to be associated with resistance to 5-FU (Chung *et al*, 2000; Murakami *et al*, 2000). Although the true mechanisms for the downregulation of OPRT expression in human cancers are still unclear, an experimental study has suggested the loss of 3q to be a genetic change responsible for the decreased OPRT activity and acquired resistance to 5-FU (Hidaka *et al*, 2003). Furthermore, the adenoviral-mediated transduction of OPRT gene was reported to result in a marked sensitisation of tumour cells to 5-FU cytotoxicity (Kanai *et al*, 1998).

Previous clinical studies also revealed the positive expression and activity of OPRT to be associated with the responsiveness to 5-FU in a variety of human cancers, including colorectal cancers (Isshi *et al*, 2002; Fujii *et al*, 2003; Ichikawa *et al*, 2003a; Matsuyama *et al*, 2006) and bladder carcinomas (Mizutani *et al*, 2004). However, these studies were performed using relatively small number of patients, and then were evaluated by biochemical assays for OPRT enzyme activity or reverse transcriptional PCR (RT–PCR) assays for OPRT gene expression. Biochemical assays are difficult to perform in clinical use, and they need a large volume of sample tissue. In the present study, we evaluated the intratumoural OPRT expression by immunohistochemistry using an anti-OPRT polyclonal antibody, obtained by immunising rabbits with the OPRT peptide. The immunochemical detection using this antibody was found to positively correlate with the OPRT enzyme activity (Sakamoto *et al*, 2005), and immunohistochemical assays are easy to perform in clinical use. Consequently, the present study demonstrated the positive expression of OPRT to be one of the significant factors for a good survival for resected NSCLC patients postoperatively treated with UFT, especially in advanced stage II to III. To our knowledge, the present study was the first clinical report using immunohistochemistry, to demonstrate the clinical significance of the OPRT expression in a relatively large number of cancer patients treated with UFT. In fact, a recent clinical study also revealed a positive OPRT activity to be associated with a better prognosis in resectable colorectal cancer patients treated with oral 5-FU (Ochiai *et al*, 2006).

On the other hand, regarding other pathways of 5-FU activation, thymidine phosphatase (TP) is considered to not be important because of the lack of its cofactor dRib-1-P at physiological concentrations (Barankiewicz and Henderson, 1977). Conversely, high TP concentration in conjunction with the normally low dRib-1-P drive the reaction in the opposite direction (Ackland and Peters, 1999). In fact, no difference was observed in the survivals of NSCLC patients treated with UFT according to the intratumoural TP expression in our pilot study by immunohistochemistry (data not shown).

Third, due to the fact that 80–90% of the administered 5-FU is degraded by DPD in humans, the first and rate-limiting enzyme of 5-FU catabolism, only a small part of the 5-FU is activated (Fischel *et al*, 1995). As a result, 5-FU degradation generally occurs in all tissues, including tumour tissues, but it reaches its highest level in the liver (Ho *et al*, 1986). In fact, a congenital deficiency of DPD can result in severe life-threatening toxicity when 5-FU is administered (Wei *et al*, 1996). In contrast, many experimental studies have demonstrated the intratumoural DPD activity to affect the resistance to 5-FU (Beck *et al*, 1994; Ishikawa *et al*, 1999). Previous clinical studies also found an overexpression of DPD in tumour cells to be associated with 5-FU resistance (Higashiyama *et al*, 2001) and a poor prognosis in many cancer patients treated with 5-FU, including NSCLCs (Huang *et al*, 2000; Nakagawa *et al*, 2002), colorectal cancers (Ichikawa *et al*, 2003b), and breast cancers (Horiguchi *et al*, 2004). Although several clinical studies reported DPD expression to not be associated with the survival of cancer patients treated with 5-FU (Ikeguchi *et al*, 2002; Kornmann *et al*, 2002; Hakamada *et al*, 2005), this discrepancy might be due to the different types of 5-FU administration such as intravenous infusion, the different antibody used, or the contamination of intratumoural stromal cells.

Based on these facts about the biomarkers associated with sensitivity to 5-FU, we thus considered a clinical study to simultaneously evaluate the expressions of TS, OPRT, and DPD, to be important for establishing the protocol of made-to-order chemotherapy for NSCLC patients. Therefore, we performed the present study using a relatively large number of patients treated with UFT. Consequently, the intratumoural expressions of TS, OPRT, and DPD were found to be independent of each other. Furthermore, a Cox multivariate analysis demonstrated all the intratumoural expressions of TS, OPRT, and DPD to be independent prognostic factors for resected NSCLC patients postoperatively treated with UFT. As a result, regarding the biomarkers associated with sensitivity to 5-FU, the survival of patients with TS-negative, OPRT-positive, and DPD-negative NSCLCs, which appeared in 12.6% (19 of 151) in the present study, was the highest among resected NSCLC patients treated with UFT.

Besides these biomarkers associated with sensitivity to 5-FU, the present study revealed that pathological stage and methods of surgical resection (a pneumonectomy *vs* other methods) were also independent prognostic factors for the resected NSCLC patients treated with UFT. Previous clinical studies also demonstrated that the survival of patients treated with a pneumonectomy is worse than that of patients treated with other surgical methods (Rocco *et al*, 1996; Ludwig *et al* 2005). On the other hand, regarding chemotherapy, most of patients with locally advanced stage II to III NSCLCs were treated with either neoadjuvant or postoperative adjuvant platinum-based chemotherapy using mitomycin/vinblastin/cisplatin or carboplatin/paclitaxel in the present study. However, we found no difference in the survivals of patients according to the platinum-based chemotherapy (data not shown). In order to clarify the clinical significance of the platinum-based chemotherapy for NSCLC patients, a randomised prospective study should thus be performed. Furthermore, among the locally advanced stage II to III cases, no difference was observed in the survivals of patients according to radiotherapy in the present study (data not shown). In fact, previous clinical studies also reported that radiotherapy itself has less survival benefit for NSCLC patients (Dautzenberg *et al*, 1999).

The efficacy of UFT is still lower in NSCLCs than in gastrointestinal cancers. For one reason, NSCLCs are human tumours with a high expression of DPD because DPD-positive tumours occurred in 45% of NSCLCs in the present study. Therefore, it is important to inhibit the DPD activity to improve the responsiveness to 5-FU against DPD-positive NSCLCs. Recently, S-1 (a combination of tegafur, gimeracil, and oteracil potassium, Taiho) has been developed for clinical use (Chu *et al*, 2004; Ichinose *et al*, 2004). Gimeracil used in S-1 is a stronger inhibitor for the intratumoural DPD activity than uracil used in UFT. As a result, S-1 might be more effective for patients with TS-negative, OPRT-positive, and DPD-positive NSCLCs, which occurred in 15.9% (24 of 151) in the present study. Therefore, patients with TS-negative and OPRT-positive NSCLCs, which appeared in 28.5% (43 of 151) in the present study, could be successfully treated by S-1 without evaluating the intratumoural DPD activity. In order to develop and simplify the protocol of made-to-order chemotherapy, a further randomised prospective study using S-1 should thus be performed to evaluate this possibility.

## Figures and Tables

**Table 1:** Patient demographics

**Patient characteristics**	**Number**	**%**
Total number of patients	151	100.0
*Age (years)*		
Median	64	
Range	35–76	
		
*Gender*		
Male	104	68.9
Female	47	31.1
		
*Smoking habitus*		
Nonsmoker	51	33.8
Smoker	100	66.2
		
*ECOG performance status*		
0	85	56.3
1	57	37.7
2	9	6.0
		
*Pathological stage*		
I	62	41.1
II	28	18.5
III	61	40.4
		
*Differentiation*		
Well	46	30.5
Moderately	54	35.7
Poorly	51	33.8
		
*Histology*		
Adenocarcinoma	84	55.6
Squamous cell carcinoma	56	37.1
Large cell carcinoma	11	7.3
		
*Method of surgical resection*		
Pneumonectomy	14	9.3
Lobectomy	123	81.5
Segmentectomy	4	2.6
Wedge resection	10	6.6
		
*Chemothrapy*		
Platinum-based chemotherapy	79	52.3
Neoadjuvant MVP	30	19.8
Neoadjuvant CBDCA/PTX	9	6.0
Postoperative adjuvant MVP	26	17.2
Postoperative adjuvant CBDCA/PTX	14	9.3
UFT	151	100.0
		
*Radiotherapy*	24	15.9

CBDCA/PTX=carboplatin/paclitaxel; ECOG=Eastern Cooperative Oncology Group; MVP=mitomycin/vinblastin/cisplatin; UFT=a combination of tegafur and uracil.

**Table 2:** Distribution of biomarkers in 151 NSCLC patients according to clinicopathological characteristics

		**TS-positive**		**OPRT-positive**		**DPD-positive**	
**Characteristics**	***n***	**(%)**	***P*-value**	**(%)**	***P*-value**	**(%)**	***P*-value**
*Smoking*							
Nonsmoker	51	52.0	0.4260	70.6	0.8411	54.9	0.1167
Smoker	100	58.8		69.0		40.0	
							
*Tumour status*							
T1, T2	102	56.9	0.3626	73.5	0.1240	40.2	0.1211
T3, T4	49	49.0		61.2		55.1	
							
*Nodal status*							
N0	88	48.9	0.1127	67.0	0.4319	38.6	0.0887
N1, N2	63	61.9		73.0		54.0	
							
*Pathological stage*							
Stage I	62	53.2	0.7905	66.1	0.4886	35.5	0.0757
Stage II	28	50.0		78.6		42.9	
Stage III	61	57.4		68.9		55.7	
							
*Differentiation*							
Well	46	60.9	0.1888	65.2	0.4395	43.5	0.9338
Moderately	54	44.4		75.9		44.4	
Poorly	51	58.8		66.7		47.1	
							
*Histology*							
Adenocarcinoma	84	42.9	0.0063	69.0	0.3170	47.6	0.5025
Squamous cell carcinoma	56	69.6		76.8		39.3	
Large cell carcinoma	11	63.6		36.4		54.5	
Neoadjuvant chemotherapy							
Neoadjuvant MVP	30	60.0	0.3700	53.3	0.0943	50.0	0.6691
Neoadjuvant CBDCA/PTX	9	33.3		77.8		33.3	
Without neoadjuvant	112	54.5		73.2		44.6	
							
*Total number of patients*	151	54.3		69.5		45.0	

CBDCA/PTX=carboplatin/paclitaxel; MVP=mitomycin/vinblastin/cisplatin; NSCLC=non-small-cell lung cancer.

**Table 3:** Multivariate regression analysis in predicting survival of NSCLC patients treated with UFT

**Variables**	**Assigned score**	**Hazard ratio**	**95%CI**	***P*-value**
*TS status*				
Negative	0	2.663	1.567–4.526	0.0003
Positive	1			
				
*OPRT status*				
Positive	0	2.543	1.508–4.287	0.0005
Negative	1			
				
*DPD status*				
Negative	0	2.840	1.685–4.786	<0.0001
Positive	1			
				
*ECOG performance status*				
0	0	1.301	0.825–2.051	0.2576
1	1			
2	2			
				
*Pathological stage*				
I	1	1.908	1.406–2.588	<0.0001
II	2			
III	3			
				
*Differentiation*				
Well	0	1.296	0.949–1.769	0.1029
Moderately	1			
Poorly	2			
				
*Method of surgical resection*				
Other methods	0	2.555	1.130–5.776	0.0242
Pneumonectomy	1			

CI=confidence interval; DPD=dihydropyrimidine dehydrogenase; NSCLC=non-small-cell lung cancer; OPRT=orotate phosphoribosyltransferase; TS=thymidylate synthase; UFT=a combination of tegafur and uracil.
